# Supplementary figures and images for: Leveraging of SARS-CoV-2 PCR Cycle Thresholds Values to Forecast COVID-19 Trends
Source: Front Med (Lausanne). 2021 Nov 1;8:743988. doi: 10.3389/fmed.2021.743988 (PMC8591051; doi:10.3389/fmed.2021.743988)

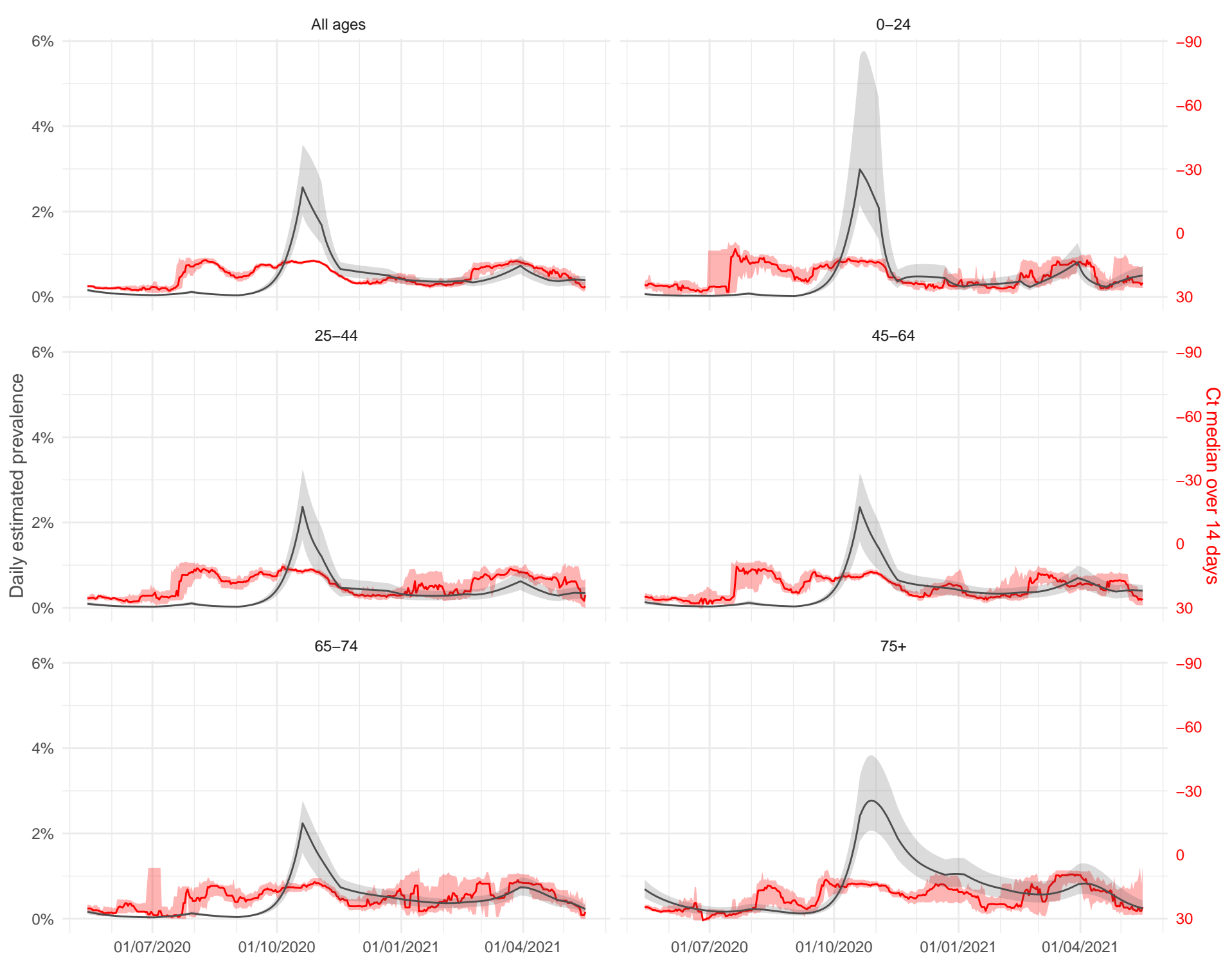

Supplement: Supplementary Figure 1 — Ct evolution by age classes with comparison to the estimated prevalence: evolution of the daily 14-day median Ct values obtained using the Abbott RealTime SARS-CoV-2 assay from April 1, 2020, to May 15, 2021 (LHUB-ULB, Brussels region, Belgium, full data figure). Reddish areas surrounding solid red curves indicate the 95% confidence interval (CI) obtained by bootstrapping method associated with daily estimates of median Ct values. Those curves are superimposed on the daily prevalence of COVID-19 infected people (proportion of infected vs. Belgian demographic data) as estimated by the age-structured extended SEIR-type mathematical model. Solid grey curves indicate the 95% CI obtained by Bayesian analysis. [file Image_1.PDF]
